# Concordance of breast cancer biomarker testing in core needle biopsy and surgical specimens: A single institution experience

**DOI:** 10.1002/cam4.4843

**Published:** 2022-06-22

**Authors:** Jessica A. Slostad, Nicole K. Yun, Aimee E. Schad, Surbhi Warrior, Louis F. Fogg, Ruta Rao

**Affiliations:** ^1^ Division of Hematology‐Oncology Rush University Medical Center Chicago Illinois USA; ^2^ Department of Internal Medicine Rush University Medical Center Chicago Illinois USA; ^3^ Division of Hematology and Medical Oncology St. Louis University St. Louis Missouri USA; ^4^ Department of Community, Systems, and Mental Health Nursing; College of Nursing Rush University Medical Center Chicago Illinois USA

**Keywords:** breast cancer, core needle biopsy, estrogen receptor, progesterone receptor, surgical pathology, tyrosine kinase‐type cell surface receptor (human epidermal growth factor receptor 2 HER2)

## Abstract

**Background:**

Accurate diagnostic biomarker testing is crucial to treatment decisions in breast cancer. Biomarker testing is performed on core needle biopsies (CNB) and is often repeated in the surgical specimen (SS) after resection. As differences between CNB and SS testing may alter treatment decisions, we evaluated concordance between CNB and SS as well as associated changes in treatment and clinical outcomes.

**Methods:**

We performed a retrospective analysis of breast cancer patients at our institution between January 2010 and May 2020. Concordance between CNB and SS was assessed for estrogen receptor (ER), progesterone receptor (PR), and human epidermal growth factor receptor 2 (HER2) by immunohistochemistry (IHC) and fluorescence in situ hybridization (FISH). Survival in patients, including recurrence, metastatic recurrence, and death, were assessed using chi‐squared likelihood ratio.

**Results:**

In total, 961 patients met eligibility criteria. Concordance, minor discordance, total concordance (concordance plus minor discordance), and major discordance between CNB and SS were reported for ER (87.7%, 9.2%, 90.8%, and 2.9%), PR (58.1%, 29.1%, 87.2%, and 12.8%), and HER2 IHC (52.5%, 20.9%, 73.4%, 26.6%), respectively. HER2 FISH concordance and major discordance were 58.5% and 1.2%, respectively. Of major discordance, ER (48.2%, *p* < 0.001) and HER2 FISH (50.0%) led to more management changes than HER2 IHC (2.4%, *p* = 0.04) and PR (1.6%, *p* = 0.10). Patients with ER major discordance had increased risk of death (6.7% concordance vs. 22.2% major discordance, *p* = 0.004).

**Conclusion:**

Overall, retesting ER and HER2 was more clinically beneficial than retesting PR. To aid decision‐making and minimize healthcare costs, we propose patient‐centered guidelines on retesting biomarker profiles.

## Graphical abstract

Retesting estrogen receptor and HER2 status provides more clinical benefit than retesting progesterone receptor status. We propose guidelines for retesting receptor profiles to aid patient care management decisions, prioritize clinical benefits, and minimize healthcare costs.

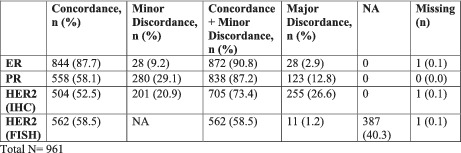

## 1 BACKGROUND

Breast cancer is the most common and second most deadly malignancy in women in the United States.^1^ Accurate diagnostic testing plays a critical role in the management of breast cancer, particularly the molecular profiling of estrogen receptor (ER), progesterone receptor (PR), and human epidermal growth factor receptor 2 (HER2) expression by immunohistochemistry (IHC) and fluorescence in situ hybridization (FISH).

Breast cancer is diagnosed by core needle biopsy (CNB) with 96–100% specificity, and molecular profiling is typically performed on CNB to assist in preoperative management decisions.^2^ The decision to administer neoadjuvant chemotherapy is based on initial CNB results. After surgical resection, the molecular profile is retested on the surgical specimen (SS), and the biomarker profile of the SS is expected to be concordant with that of the CNB. Retesting guidelines are often determined by individual institutions and providers in the United States. As discordance between CNB and SS biomarker profiles has been reported, some clinicians favor retesting receptor status on all SS to avoid overlooking potentially beneficial therapy options. Possible causes of discordance include changing tumor characteristics, intratumoral heterogeneity, and sampling and analytical errors.^3^, ^4^ The prevalence of CNB and SS discordance is unknown, and it is unclear whether discordance leads to changes in management that justify the cost of additional testing. Importantly, the clinical impact of discordance on long‐term morbidity and mortality in breast cancer requires further investigation.

The American Society of Clinical Oncology (ASCO) and the College of American Pathologists (CAP) established a list of clinical considerations in 2013 (updated in 2018) for retesting HER2 status in the SS after CNB based on suspicion of discordance per the National Comprehensive Cancer Network (NCCN) guidelines.^5^, ^6^, ^7^ These guidelines recommend retesting the SS if the CNB indicated an infiltrating ductal or lobular carcinoma or a 90% pure tubular, mucinous, cribriform, or adenoid cystic carcinoma that was HER2‐positive, ER/PR‐positive, and grade 1 histologically. The guidelines also recommend retesting HER2 status on the SS if the initial CNB is HER2‐negative in tumors that are grade 3, has a minor level of invasive disease, shows morphological discrepancy from the SS, or if there is concern regarding CNB handling.^7^ The updated 2020 ASCO/CAP *Guidelines for Estrogen and Progesterone Receptor Testing in Breast Cancer* suggest retesting ER in cases of highly unusual ER‐negative or ER‐positive results.^7^, ^8^ Highly unusual ER‐negative results include low‐grade invasive carcinomas; classic lobular carcinoma; pure tubular, cribriform, or mucinous carcinoma; or encapsulated or solid papillary carcinomas. Highly unusual ER‐positive results include metaplastic carcinomas, adenoid cystic carcinoma or other salivary gland‐like carcinomas, secretory carcinoma, or carcinomas with apocrine differentiation.^7^ Criteria for possible PR discordance were not addressed. Retesting criteria were established based on systematic literature review and factors indicative of discordance; however, these guidelines were derived from lower levels of supportive evidence when compared to existing recommendations. For comparison, international guidelines recommend biomarker assessment on either CNB or SS. The NCCN guidelines do not represent absolute indications for retesting. Ultimately, the decision to retest is based on the judgment of the clinician or the institutional policy in the United States.

Several studies worldwide have assessed the concordance of receptor testing in breast cancer with mixed conclusions.^2^, ^9^ Beyond differences in receptor status, the clinical significance of discordance is not well understood.^10^ There is a need to better understand the discrepancy between CNB and SS testing as well as the utility of retesting. Defining treatment changes in response to discordant results would clarify whether retesting is worth the cost. Given the high prevalence of breast cancer in women, retesting has substantial cost and clinical implications for the individual and the healthcare system.^1^ We sought to define the risk of discordance between CNB and SS, and, more importantly, the clinical consequences of discordance, as this has implications for patients, clinicians, and the healthcare system.

## 2 METHODS

We performed a retrospective analysis of patients with histologically confirmed invasive breast cancer on CNB and SS pathology at our institution between January 2010 and May 2020. This study was conducted in accordance with the Rush University Medical Center Institutional Review Board (IRB) and the Declaration of Helsinki. Patients ≥18 who underwent primary surgical resection or had neoadjuvant chemotherapy with residual disease on surgical excision were included in the study. Patients were included if pathology with receptor expression testing (ER, PR, and HER2 IHC and/or FISH) was available on both CNB and SS. All testing was performed at Rush University Medical Center. ER and PR were lab developed tests (LDT, Invitro Diagnostics), which is a CAP accredited, internally validated test. Biomarker tests are automated. Time to fixation and time in formalin was routinely included after the updated 2013 ASCO‐CAP Guidelines. HER2 IHC was evaluated by HER‐2/neu (HercepTest) and FISH by PathVysion assay kit by Abbott Inc.

Exclusion criteria included patients with CNB or SS obtained at outside institutions, pathologic complete response on SS, and patients with distant metastatic disease. Patients with distant metastatic disease were excluded from the analysis given biomarker differences between the primary tumor and metastatic disease sites has different management implications than in the neoadjuvant or adjuvant setting. Medical records were individually reviewed to ensure data accuracy.

Concordance rates between CNB and SS were evaluated for ER, PR, and HER2 IHC and FISH. ER and PR status were defined per NCCN guidelines as “positive,” “low‐positive (ER),” or “negative” based on IHC. The major discrepancy was defined as a change in receptor status. The minor discrepancy was defined as >10% change in expression with no change in receptor status. Total concordance included both concordance and minor discordance. For HER2, major discordance was defined as a change in receptor status (e.g., positive or negative) based on IHC or FISH. The minor discrepancy was defined as a change in HER2 IHC (0–3) or FISH amplification without a change in receptor status.

The frequency of treatment changes in patients with discordant biomarker expression was assessed. Patients with major discordance were reviewed by an investigator to determine whether the treatment course was changed based on the SS receptor profile (for example, recommendation for or against hormone or HER2 directed therapy). A second independent reviewer was available to resolve discrepancies in the data. Statistical analysis was performed with chi‐squared likelihood ratio using SPSS (IBM Corporation) software. P values were performed by Pearson's chi‐squared test or Fisher's exact test. Recurrence, metastatic recurrence, and death from any cause were evaluated for correlation of concordance, minor, or major discordance. *p*‐values were assessed by Fisher’s exact test.

We evaluated the impact of retesting on nationwide healthcare expenditure. The number of concordance tests that could have been safely omitted was analyzed for projected cost savings in the United States in 2020.^1^, ^38^

Finally, we performed a literature search from July 2020 to September 2020 to identify previously conducted studies of concordance between CNB and SS. Eligible studies were identified using a MEDLINE search using the keywords including “Breast Cancer,” “Estrogen Receptor (ER),” “Progesterone Receptor (PR),” “HER2 or HER2/neu,” “Concordance,” “Discordance,” “Core Needle Biopsy,” “Surgical Specimen,” “Excisional Biopsy,” and “Immunohistochemistry.” We excluded meta‐analyses, studies examining receptor status on non‐breast metastatic sites or non‐invasive breast cancer, and studies that did not report specific CNB and SS concordance rates for ER, PR, or HER2.

## 3 RESULTS

Of 5581 breast cancer patients who underwent treatment at Rush University Medical Center between January 2010 and May 2020, 961 patients met eligibility criteria, and 86 of these patients received neoadjuvant therapy. Baseline characteristics were assessed for the total cohort (Table 1). Concordance, minor discordance, total concordance (concordance + minor discordance), and major discordance were assessed for ER, PR, and HER2 by IHC and FISH for all patients (Table 2) and patients with neoadjuvant therapy (Table 3). Concordance was highest for ER (87.7% in all patients, 80.2% in neoadjuvant cohort). Major discordance was most common in HER2 IHC (26.6% in all patients, 29.1% in neoadjuvant cohort) and minor discordance highest in PR (29.1% in all patients, and 23.3% in neoadjuvant cohort).

**TABLE 1:** Baseline patient characteristics

Baseline characteristics	
**Age (diagnosis)**	
Mean	63
≤50	158 (16.4)
>50	803 (83.5%)
**Gender**	
Female	953 (99.1%)
Male	7 (0.7%)
Unknown/Not reported	1 (0.1%)
**Race**	
White	515 (53.5%)
Black or African American	323 (33.6%)
Asian	24 (2.5%)
American Indian/Alaska Native	2 (0.2%)
Native Hawaiian or Other Pacific Islander	1 (0.1%)
More than one race	4 (0.4%)
Unknown/Not Reported	93 (9.7%)
**Ethnicity**	
NOT Hispanic or Latino	853 (88.7%)
Hispanic or Latino	99 (10.3%)
Unknown/Not Reported	9 (0.9%)
**Histology ‐ SS**	
Invasive ductal carcinoma	791 (82.2%)
Invasive lobular	125 (13.0%)
Invasive mucinous carcinoma	20 (2.1%)
Papillary carcinoma	4 (0.4%)
Neuroendocrine features	4 (0.4%)
Apocrine carcinoma	1 (0.1%)
Invasive secretory carcinoma	1 (0.1%)
Invasive tubular carcinoma	2 (0.2%)
Mixed features	11 (1.1%)
Other	3 (0.3%)
**Tumor grade: SS**	
1	167 (17.4%)
2	451 (46.9%)
3	308 (32.0%)
**Tumor size: SS** (mm)	
≤20	696 (72.3%)
>20 and ≤ 50	225 (23.4%)
>50	35 (3.6%)
**HER2 (IHC): CNB**	
0	163 (17.0%)
1+	425 (44.2%)
2+	333 (34.7%)
3+	40 (4.2%)
**HER2 (IHC): SS**	
0	294 (30.6%)
1+	449 (46.7%)
2+	176 (18.3%)
3+	42 (4.4%)
**Neoadjuvant**	
Yes	86 (8.9)
No	875 (89.5%)
**Neoadjuvant treatment**	
Hormone therapy	6 (6.9%)
Systemic chemotherapy	71 (81.6%)
HER2 directed therapy	9 (10.3%)
Other	1 (1.1%)
**Adjuvant**	
Yes	849 (88.3%)
No	65 (6.8%)
**Radiation**	
Yes	585 (60.9%)
No	348 (36.2%)

Abbreviations: CNB, core needle biopsy; SS, surgical specimen, HER2, human epidermal growth factor receptor 2, IHC, immunohistochemistry.

**TABLE 2:** Concordance, minor discordance, major discordance in ER, PR, HER2 IHC, and HER2 FISH in all patients

	Concordance, *n* (%)	Minor discordance, *n* (%)	Concordance + minor ddiscordance, *n* (%)	Major discordance, *n* (%)	NA	Missing (*n*)
**ER**	844 (87.7)	28 (9.2)	872 (90.8)	28 (2.9)	0	1 (0.1)
**PR**	558 (58.1)	280 (29.1)	838 (87.2)	123 (12.8)	0	0 (0.0)
**HER2 (IHC)**	504 (52.5)	201 (20.9)	705 (73.4)	255 (26.6)	0	1 (0.1)
**HER2 (FISH)**	562 (58.5)	NA	562 (58.5)	11 (1.2)	387 (40.3)	1 (0.1)

Total *N* = 961.

Abbreviations: ER, estrogen receptor, PR, progesterone receptor, HER2, human epidermal growth factor receptor 2, IHC, immunohistochemistry, FISH, fluorescence in situ hybridization; N, number; NA, Not assessed.

**TABLE 3:** Concordance, minor discordance, major discordance in ER, PR, HER2 IHC, and HER2 FISH in the neoadjuvant cohort

	Concordance, *n* (%)	Minor discordance, *n* (%)	Concordance + minor discordance, *n* (%)	Major discordance, *n* (%)	NA	N
**ER**	69 (80.2)	8 (9.3)	77 (89.5)	9 (10.5)	0	86
**PR**	44 (51.2)	20 (23.3)	64 (74.4)	22 (25.6)	0	86
**HER2 (IHC)**	48 (55.8)	13 (15.1)	61 (70.9)	25 (29.1)	0	86
**HER2 (FISH)**	50 (58.1)	NA	50 (58.1)	4 (4.7)	32 (37.2)	86

Abbreviations: ER, estrogen receptor; FISH, fluorescence in situ hybridization; HER2, human epidermal growth factor receptor 2; IHC, immunohistochemistry; PR, progesterone receptor; N, number; NA, Not assessed.

Although discordance was more common in PR and HER2 IHC than in ER and HER2 FISH biomarker profiles, major discordance leading to treatment changes was more common in ER and HER2 FISH. Of major discordance in the total cohort (961 patients), ER (48.2%, *p* < 0.001) and HER2 FISH (50.0%) led to more changes in management than HER2 IHC (2.4%, *p* = 0.04) and PR (1.6%, *p* = 0.10) (Table 4, Table S1). Retesting HER2 IHC/FISH did not change management when the initial CNB was HER2 positive. In the neoadjuvant cohort (*n* = 86), discordance leading to treatment changes represented a small group of patients and did not show statistical significance (Table S2).

**TABLE 4:** Treatment changes based on discordance in ER, PR, HER2 IHC, and HER2 FISH in all patients

	Major discordance (*n*, %)	Minor discordance (*n*, %)	*p*‐value*
**ER (total *n*)**	28	88	<0.001
No	14 (51.2)	87 (100)	
Yes	13 (48.2)	0 (0)	
Missing	1	1	
**PR (total *n*)**	123	281	0.10
No	121 (98.4)	271 (100)	
Yes	2 (1.6)	0 (0)	
Missing	0	9	
**HER2 IHC (total n)**	255	201	0.04
No	249 (97.7)	201 (100)	
Yes	6 (2.4)	0 (0)	
Missing	0	0	
**HER2 FISH (total n)**	11	NA	
No	5 (50.0)		
Yes	5 (50.0)		
Missing	1		

Abbreviations: ER, estrogen receptor, PR, progesterone receptor, HER2, human epidermal growth factor receptor 2, IHC, immunohistochemistry, FISH, fluorescence in situ hybridization; N, number; NA, Not assessed.

^a^ Fisher's exact test.

Recurrence, metastatic recurrence, and death from any cause were assessed for ER and HER2 IHC based on concordance and discordance (Tables S3 and S4). PR was not assessed given that clinically significant treatment changes were uncommon, and HER2 FISH was omitted from the descriptive analyses for low incidence of major discordance. There was a statistically significant increase in death from any cause in ER major discordance (*n* = 6, 22.2%, *p* = 0.004) compared to concordance (*n* = 54, 6.7%) or minor discordance (*n* = 11, 12.9%) (Table S3). Recurrence in ER concordant, minor discordant, and major discordant specimens was noted in 8.1%, 8.2%, and 15.4% of patients (*p* = 0.38), respectively. Metastatic recurrence was noted in 22.6%, 31.6%, and 50.0% of patients (*p* = 0.19) with ER concordance, minor discordance, and major discordance, respectively (Table S3). In HER2 IHC, there was a non‐statistically significant trend to increased recurrence and metastatic recurrence in patients with discordant compared to concordant samples (Table S4). Death from any cause was highest in HER2 IHC with minor discordance (*n* = 20, 10.6%) compared to specimens with concordance (*n* = 41, 8.5%) and major discordance (*n* = 10, 4.0%) (*p* = 0.02, Table S4).

The incidence of recurrence, metastatic recurrence, and death from any cause were assessed in patients who underwent treatment changes based on discordance. Patients whose treatment changed based on major discordance overall trended towards worse outcomes; however, this was not statistically significant (Table S5). Although not statistically significant, patients with ER discordance‐driven treatment changes tended to have higher incidences of recurrence (8.6% vs. 16.7%, *p* = 0.30), metastatic disease at recurrence (31.8% vs. 50.0%, *p* > 0.99), and death from any cause (14.3% vs. 16.7%, *p* = 0.69) compared to patients with no treatment changes. In patients with HER2 IHC discordance‐driven treatment changes, there was a trend towards increased risk of recurrence (7.4% vs. 16.7%, *p* = 0.38) but not towards increased risk of metastatic disease (21.0% vs. 0%, *p* > 0.99) or death (6.9% vs. 0%, *p* > 0.99) compared to patients with no treatment changes. In summary, we did not find a statistically significant increase in the risk of recurrence, metastatic recurrence, or death due to treatment changes arising from ER or HER2 IHC discrepancies between CNB and SS in our cohort (**Table** **S5**).

**TABLE 5:** Studies evaluating retesting biomarkers on surgical specimens

Author (publication year)	Years collected	*N*	*N* in primary surgical cohort	*N* in neoadjuvant cohort	Primary surgical cohort concordance rate % (kappa value)				Neoadjuvant cohort concordance rate % (kappa value)				Country	Recommend retesting?	*N* patients with management changes
					ER	PR	HER2		ER	PR	HER2				
							IHC alone	FISH after IHC			IHC	FISH after IHC			
**Mann (2005)**	1999–2002	100	100		86.0 (N/A)	83.0 (N/A)	42.0 (N/A)						Australia	Yes	9
**Ozdemir (2007)**	2001–2005	199	199		90 (N/A)	86.7 (N/A)	79.3 (N/A)						Turkey	No	
**Abdsalah et al (2008)**	1998–1999, 2001–2002	129	129		96.9 (N/A)	84.5 (N/A)							Sweden	No	
**Arnedos et al (2009)**	2005–2007	336	336		98.2 (N/A)	85.0 (N/A)		98.8 (N/A)					United Kingdom		
**Park et al (2009)**	2003–2005	104	104		99.0 (0.977)	97.1 (0.94)	86.5 (0.881)						South Korea	No	
**Tamaki et al (2010)**	2002–2009	353	353		92.9 (0.82)	77.9 (0.66)	89.3 (0.64)						Japan	Yes	
**Uy et al (2010)**	2003–2008	160	160		86.2 (N/A)	92.7 (N/A)							United States, Philippines	Yes, if doubt in adequate testing	
**Lorgis et al (2011)**	2005–2006	175	175		84.0 (N/A)	78.3 (N/A)		98.3 (N/A)					France	Yes	
**Ough et al (2011)**	2011	209	209		88.0 (0.7087)	78.0 (0.5425)	81.0 (0.5908)						United States	Yes	
**Ricci et al (2012)**	2011	69	69		95.0 (0.89)	87 (0.70)	78.0 (0.61)						Brazil	Yes	
**Chen et al (2013)**	2009–2012	298	298		93.6 (0.827)	85.9 (0.704)		96.3 (0.894)					China		
**Greer et al (2013)**	2009–2011	208	208		89.0 (0.56)	89.0 (0.71)	93.0 (0.63)						United States	Yes, for HER2	
**Dekker et al (2013)**	2006–2008	122	122		99.1 (0.966)		82.4 (0.505)*						Netherlands	Yes, if ER negative on CNB.	
**Motamedolshariati et al (2014)**	2009–2011	30	30		96.7 (0.93)	90.0 (0.79)	93.3 (0.857)						Iran		
**Munch‐Petersen et al (2014)**	2014	89	89		98.0 (1.00)		84.0 (N/A)	95.4 (N/A)					Denmark	Case‐by‐case	
**Vohra et al (2016)**	2002–2014	134	134		96.2 (N/A)	77.5 (N/A)		96.74 (N/A)					United States	Needs further research	
**Asogan et al (2017)**	2005–2012	560	560		96.1 (N/A)	89.1 (N/A)	96.8 (N/A)						Singapore	Yes if CNB triple negative.	
**Chen et al (2017)**	2007–2015	1003	1003		78.8 (0.522)	73.5 (0.441)	62.6 (0.451)						China	Yes	16.60%
**Ensani et al (2017)**	2011–2014	100	100		90.0 (N/A)	81.0 (N/A)	97.3 (N/A)						Iran		
**Kombak et al (2017)**	2011–2015	284	284		93.3 (N/A)	89.4 (N/A)	90.1 (N/A)						Turkey	Yes if negative CNB	
**Meattini et al (2017)**	2014–2015	101	101		94.1 (0.82)	88.1 (0.60)	84.5 (0.74)						Italy		
**Clark et al (2018)**	N/A	99	99		99.0 (N/A)	95.0 (N/A)							United States	Yes	
**Jeong et al (2019)**	2014–2017	629	629		96.5 (0.883)	93.0 (0.824)	81.4 (0.591)	99.7 (0.988)					South Korea		
**Khoury et al (2011)**	2011	176	169	7	93.0 (0.78)	90.0 (0.76)	94.0 (0.79)		No separate analysis performed for neoadjuvant cohort				United States	Yes	
**You et al (2017)**	2014	1371	1219	152	96.7 (0.903)	94.3 (0.870)	84.8 (0.684)		92.9 (0.858)	88.2 (0.746)	86.6 (0.762)	N/A	South Korea		
**Robertson et. al (2019)**	2016–2017	716	526	190	98.6 (0.917)	89.3 (0.725)	75.4 (0.462)		96.2 (0.887)	73.9 (0.490)	76.7 (0.539)	93.8 (0.757)	Sweden	Yes, for HER1 and Ki‐67	
**Berghuis et al (2019)**	2016–2018	8881	7858	1023	96.3 (N/A)	86.71 (N/A)	99.53 (N/A)		91.15 (N/A)	76.43 (N/A)	95.0 (N/A)	N/A	Netherlands	Needs further research	

Abbreviations: CNB, core needle biopsy; ER, estrogen receptor; FISH, fluorescence in situ hybridization; HER2, human epidermal growth factor receptor 2; IHC, immunohistochemistry; N/A, not available; N, Number; PR, progesterone receptor.

^**a**^ Concordance rate of 82.4 (0.505) when HER2 was evaluated using three scores. Concordance rate of 96.2 (0.813) when HER2 was evaluated as a dichotomous variable (positive or negative).

### 3.1 Cost analysis

Of the 961 patients in our cohort, 259 (26.95%) demonstrated complete concordance between CNB and SS for ER, PR, and HER2 IHC. For 2020, the American Cancer Society estimated that 276,480 new cases of invasive breast cancer would be diagnosed in women in the United States.^1^ Using the complete concordance rate calculated at our institution and the projected number of new cases nationwide, we estimated that 74,511 patients received unnecessary SS retesting. We extrapolated our institution's estimated cost of retesting ER, PR, and HER2 IHC and projected an increased cost of more than $43 million dollars in the United States for 2020 alone.

## 4 DISCUSSION

As breast cancer is the most common malignancy in women, retesting SS for breast cancer patients has important implications for individuals and the healthcare system in the United States.^1^ At our institution, total concordance for all patients (concordance and minor discordance) was 90.8%, 87.2%, 73.4%, and 58.5% for ER, PR, HER2 IHC, and HER2 FISH, respectively (Table 2). There was major discordance of 2.9%, 12.8%, 26.6%, and 1.2% for ER, PR, HER2 IHC, and HER2 FISH, respectively (Table 2). Although major discordance was more common in PR and HER2 IHC than in ER or HER2 FISH biomarker profiles, major discordance leading to treatment changes was more common in ER (48.2%) and HER2 FISH (50.0%). Importantly, the concordance rates observed in this study are similar to rates reported in the literature **(**Table 5 **).**

There are various explanations for discordance between CNB and SS biomarker profiles in breast cancer, including tumor heterogeneity and pre‐analytic variation.^11^ It is also thought that ER/HER2 activity can be lost during the time between tumor acquisition and fixation. ASCO/CAP addressed factors that influence variability in ER, PR, and HER2 IHC, such as specimen handling, tissue fixation, and analytical testing methods, and thresholds for interpretation of positive/negative results.^5^, ^6^, ^7^ Although these guidelines help to mitigate variation in receptor biomarkers, they do not offer standardized retesting guidelines.

Retesting HER2 on SS did not lead to a change in management if HER2 was positive on initial CNB; however, we must also consider cases in which HER2 was equivocal on initial CNB (Table S1). Gupta et al. (2019) compared overall survival and disease‐free survival in patients with equivocal HER2 FISH and found no statistically significant difference in survival between patients treated with trastuzumab and untreated patients.^12^ When systemic treatment modalities for patients with HER2‐negative and HER2 equivocal malignancies were compared, there was no statistically significant difference in treatment decisions. These investigators concluded that adjudication of equivocal HER2 results into positive or negative categories did not alter treatment decisions or impact patient outcomes.^12^

### 4.1 Patient outcomes for recurrence, metastatic recurrence, or death from any cause

Recurrence, metastatic disease at recurrence, and death from any cause were assessed for ER and HER2 IHC based on concordance, minor discordance, and major discordance. Patients with major discordance or those with treatment changes tended to have worse outcomes; however, this was only statistically significant for ER major discordance and death from any cause. These findings suggest that ER discordance, but not HER2 IHC discordance, correlates to an increased risk of mortality in patients. Instead, HER2 IHC demonstrated a statistically significant increase in mortality in patients with concordance (8.5%) and minor discordance (28.2%) compared to major discordance (4.0%). This may be due to the lower number of treatment changes associated with HER2 IHC/FISH compared to ER. HER2‐positive patients also tended to be treated with HER2 directed therapy, which may differentially influence survival. The poor survival or recurrence in patients with ER discordance may have several explanations. Low‐positive ER tumors may behave more similarly to triple negative disease, which tends to have worse outcomes compared to patients with strongly positive ER expression. In addition, tumors with high heterogeneity may be at risk of discordant results and respond less robustly to therapy compared to tumors with high ER expression. Clinicians should be aware that discordance may be a poor prognostic risk factor for patients with breast cancer.

### 4.2 Literature review

In total, 27 reports evaluating biomarker receptor concordance were included in our literature review (Table 5).^2^, ^9^, ^13^, ^14^, ^15^, ^16^, ^17^, ^18^, ^19^, ^20^, ^21^, ^22^, ^23^, ^24^, ^25^, ^26^, ^27^, ^28^, ^29^, ^30^, ^31^, ^32^, ^33^, ^34^, ^35^, ^36^, ^37^ Studies included worldwide populations with cohort sizes ranging from 30 to 8881. All studies included ER expression, 25 included PR expression, 24 included HER2 data analysis, and four included a neoadjuvant cohort. Overall, PR tended to have lower rates of concordance than ER or HER2 IHC, which is similar to our findings. The majority of the studies did not include HER2 FISH. Conclusions on retesting SS were mixed and often advised a need for further research. Fourteen studies recommended retesting in certain circumstances, three recommended against retesting, and 10 either did not comment, recommended case‐by‐case decisions, or cited a need for further research. As our rates of concordance were consistent with prior findings, we used our results to propose best practice guidelines for retesting SS.

### 4.3 Proposed best practice guidelines

Our institution adopted best practice guidelines for retesting receptor expression on SS (Table 6). In patients with ER >5%, we found that retesting SS did not lead to clinically relevant management changes, because clinicians often chose to continue anti‐estrogen therapy. However, in cases where ER was negative or low‐positive (more frequent in ER 1–5% than 5–10%), discordance between CNB and SS was more common and led to management changes. Therefore, we recommend retesting SS if ER is negative (0%). If ER is low‐positive (1–5%), retesting is considered if the clinician would change their management based on discordant results. PR discordance, although more common, did not have a clinical impact on decision‐making. For HER2 IHC/FISH, we do not recommend retesting if the CNB was HER2 positive and the patient is undergoing HER2 directed therapy, because clinical management rarely changes based on the SS results. In patients with HER2 amplified or non‐amplified FISH, we recommend retesting if the clinician would change management based on discordant results (i.e., the clinician would consider not giving HER2 directed therapy). Given the incidence of discordance in HER2 IHC/FISH, we suggest that retesting is reasonable if clinical suspicion of discordance exists. In situations where discordant results arise from CNB and retested SS, the use of genomic assays such as Oncotype DX and MammaPrint can help clinicians guide therapy. These best practice guidelines could help clinicians to determine the necessity of retesting the SS when considered alongside the NCCN guidelines on pathologic features of discordance.

**TABLE 6:** Proposed patient‐centered best practice guidelines for retesting surgical specimens after core needle biopsy

**Proposed patient‐centered, best practice guidelines for retesting surgical specimens after core needle biopsy**
Estrogen receptor*
If 0% (negative), retest primary surgical specimen and patients with neoadjuvant chemotherapy.
If 1–5% (low‐positive), consider retesting surgical specimen (primary and neoadjuvant) if disease management would be altered by addition of hormone therapy or if clinical suspicion of discordance exists.
If >5%, do not test surgical specimen unless suspicious of discordance.
Progesterone receptor*
If PR 0% (negative) AND ER 0% (negative), retest surgical specimen.
If PR 0% (negative) AND ER 1–5% (low‐positive), consider retesting surgical specimen if disease management would be altered by addition of hormone therapy or if clinical suspicion of discordance exists.
If PR 0% (negative) AND ER >5% (low‐positive), do not retest surgical specimen unless suspicious of discordance.
If PR ≥1% (positive) AND ER >5%, do not retest surgical specimen unless suspicious of discordance.
HER2 IHC/ FISH amplification**
If IHC 3+ or 2+ FISH amplified and patient undergoing neoadjuvant HER2 directed therapy, do not retest surgical specimen unless disease management would be altered or clinical suspicion for discordance exists.
If IHC 3+ or 2+ FISH amplified without neoadjuvant treatment or FISH non‐amplified, consider retesting surgical specimen if disease management would be altered or if clinical suspicion for discordance exists.
If IHC 0–1+, do not retest unless disease management would be altered or clinical suspicion for discordance exists.
*Clinical and pathologic features associated with possible ER/PR discordance^7^, ^8^: **Unusual Negative ER**: Low‐grade invasive ductal carcinoma; lobular carcinoma; pure tubular, cribriform, or mucinous carcinomas; encapsulated or solid papillary carcinomas **Unusual Positive ER**: Metaplastic carcinomas, adenoid cystic carcinoma or other salivary gland‐like breast carcinomas, apocrine differentiation **Clinical and pathologic features associated with possible HER2 discordance^6^: **Unusual Negative HER2**: Grade 3 tumor, small invasive tumor, morphologically distinct histology, equivocal ISH and IHC testing, doubt of handling of CNB specimen, insufficient sample, concern for heterogeneity in high grade cancer, or pathologist suspicion for testing error **Unusual Positive HER2**: Grade 1 carcinoma with infiltrating ductal/ lobular carcinoma (ER/PR positive); tubular, mucinous, cribriform, or adenoid cystic (ER/PR negative) carcinomas *** All cases concerning for discordance are recommended to have consultation and review by breast cancer specialized pathologist, if available.

Abbreviations: ER, estrogen receptor; FISH, fluorescence in situ hybridization; HER2, human epidermal growth factor receptor 2, IHC, immunohistochemistry; FISH, fluorescence in situ hybridization; PR, progesterone receptor.

### 4.4 Cost implications

Similar to the study by VandenBussche et al., our institution's estimated cost of retesting ER, PR, and HER2 (IHC) projected an annual cost upwards of $43 million in the United States.^38^ Our projected costs are likely underestimated due to the difficulty of accounting for the indirect costs of SS retesting, such as the time and productivity spent performing the tests and interpreting the results. This estimation was made without considering patient‐specific indications for retesting or the small percentage of patients receiving neoadjuvant therapy.

### 4.5 Strengths and limitations

We performed a large, retrospective analysis of receptor concordance between primary surgical and neoadjuvant breast cancer specimens comparable to others reported in the literature. Our study focused on clinically relevant end points based on discordance, including treatment changes and recurrence, metastatic recurrence, and death from any cause. Moreover, our data was collected from an academic and tertiary referral hospital in Chicago, a city with a diverse patient population. It is thought that receptor expression rates can be influenced by population‐dependent variables (i.e., age, race, birth rate, etc.). Previous studies have looked at discordance rates primarily in Asian, European, and Middle Eastern populations (Table 6). The diversity present in our cohort increases the generalizability of our results to the population in the United States.

Our study also has inherent limitations given its retrospective design. All data were extracted from medical records (clinical notes and pathology reports), and incomplete or missing werea was excluded. Our study spans a 10‐year period, and the recent advent of tumor profiling and genomic sequencing has enabled clinicians to make and alter therapy decisions based on predicted response to certain therapies, adding another layer of complexity to the treatment decision process. In addition, we did not have the resources to contact each patient in the data set to confirm recurrence and outcome status. There are potential confounding factors to the survival analysis, such as the time from initial diagnosis to recurrence, metastatic disease, or death. In addition, biological factors could also influence survival given Ki67 was not assessed and both ER‐positive, low positive, and negative cases were included in the concordant and discordant cohorts. Therefore, the interpretation of our survival data may not reflect true outcomes. Finally, death from any cause does not imply causation, as multiple factors play a role in mortality of breast cancer patients.

## 5 CONCLUSIONS

Although discordance was more common in PR and HER2 IHC than in ER or HER2 FISH biomarker profiles, major discordance leading to treatment changes was more common in ER and HER2 FISH. Our findings suggest that retesting ER and HER2 is more clinically beneficial than retesting PR. When discordance was present between the CNB and SS, clinical outcomes tended to be poor but did not reach statistical significance. We did find a statistically significant association between death from any cause and cases of ER major discordance. Our study suggests that retesting ER and HER2 can be clinically beneficial. Clinicians should limit retesting to cases in which discordance would change clinical management. Our proposed best practice guidelines promote a patient‐centered approach to breast cancer care that minimizes patient and healthcare costs.

## AUTHOR CONTRIBUTION STATEMENT

All authors have reviewed, discussed, and agreed to their individual contributions. Jessica Slostad: Conceptualization, data curation, formal analysis, investigation, methodology, project administration, supervision, validation, visualization, writing – original draft, and writing – review and editing. Nicole Yun: Data curation, methodology, formal analysis, investigation, visualization, writing – original draft (including cost analysis), and writing – review and editing. Aimee Schad: Data curation, investigation, visualization, writing – original draft, and writing – review and editing. Surbhi Warrior: Data curation, investigation, visualization, writing – original draft, and writing – review and editing. Louis Fogg: Formal analysis, methodology, validation, and writing – review and editing. Ruta Rao: Conceptualization, formal analysis, supervision, validation, visualization, and writing – review and editing.

## FUNDING INFORMATION

No outside or third‐party funding was used. Internal funding through the Rush Research Mentoring Program was used for manuscript editing services.

## DISCLOSURES/DISCLAIMERS

The authors do not have any disclosures or conflicts of interest related to this manuscript to report.

## ETHICS STATEMENT

This study was conducted in accordance with the Rush University Medical Center Institutional Review Board (IRB) and the Declaration of Helsinki.

## Supporting information


Table S1
Table S2:
Table S3:
Table S4:
Table S5:

## Data Availability

The data that support the findings of this study are available from the corresponding author upon reasonable request.
