# Supplementary material for: Concordance of breast cancer biomarker testing in core needle biopsy and surgical specimens: A single institution experience
Source: Cancer Med. 2022 Jun 22;11(24):4954–65. doi: 10.1002/cam4.4843 (PMC9761085; doi:10.1002/cam4.4843)
Supplement: Supplementary file 1 — Table S1 Table S2: Table S3: Table S4: Table S5: [file CAM4-11-4954-s001.zip › CAM4_4843_Table 1 supplemental JAS clean.docx]

**Patient Characteristics of Major Discrepancies Leading to Treatment Changes**

| **ER/PR Major Discrepancies** | | | | | | | | | | | | | | | | | | | | | | |
| --- | --- | --- | --- | --- | --- | --- | --- | --- | --- | --- | --- | --- | --- | --- | --- | --- | --- | --- | --- | --- | --- | --- |
| **Case ID** | **Age** | **Race** | **Ethnicity** | **Diag-nosis** | **ER- CNB** | **ER-SS** | **PR-CNB** | **PR-SS** | **HER2 IHC-CNB** | **HER2 IHC-SS** | **FISH-CNB** | **FISH-SS** | **Tumor size (mm)-SS** | **Tumor Grade-SS** | **Stage** | **Neo-adjuvant Treatment** | **Adjuvant Treatment** | **Treatment Impact** | **Rad-iation** | **Re-currence** | **Metastatic disease at recurrence?** | **Death** |
| 1 | 61 | Caucasian | NOT Hispanic or Latino | IDC | 0% | 2% | 2% | 0% | 1+ | 0 | Non-amplified | Non-amplified | 26 | 3 | pT2N0(sn) | Systemic Chemotherapy | Hormonal therapy | Hormonal therapy | yes | yes | no | no |
| 2 | 67 | Unknown / Not Reported | Hispanic or Latino | IDC | <1% | 5% | 0% | 0% | 1+ | 2+ | N/A | Non-amplified | 16 | 2 | pT1cN0sn |  | Systemic Chemotherapy; Hormone therapy | Hormonal therapy | yes | no |  | no |
| 3 | 53 | Black or African American | NOT Hispanic or Latino | IDC | 0% | 2% | 0% | <1% | 1+ | 1+ | Non-amplified | Non-amplified | 19 | 3 | pT1cN2a | Systemic Chemotherapy | Hormonal therapy | Hormonal therapy | yes | no |  | yes |
| 4 | 34 | Unknown / Not Reported | NOT Hispanic or Latino | IDC | 0% | 5% | 0% | 5% | 0 | 1+ | Non-amplified | Non-amplified | 22 | 3 | pT2N0(sn) |  | Systemic Chemotherapy; Hormone therapy | Hormonal therapy | no | no |  | no |
| 5 | 52 | Black or African American | NOT Hispanic or Latino | IDC | 0% | 20% | 0% | 0% | 1+ | 0 | N/A | Non-amplified | 11 | 3 | pT1cN0 |  | Systemic Chemotherapy; Hormone therapy | Hormonal therapy | no | no |  | no |
| 6 | 72 | Black or African American | NOT Hispanic or Latino | ILC | 0% | >90% | 0% | 0% | 0 | 1+ | N/A | N/A | 5 | 2 | pT1aNx |  | Hormonal therapy | Hormonal therapy | no | no |  | no |
| 7 | 59 | Caucasian | Hispanic or Latino | IDC | <1% | 1% | 0% | 0% | 3+ | 3+ | Amplified | Amplified | 20 | 2 | pT1cN1a(sn) |  | Systemic chemotherapy; HER2 therapy; Hormone Therapy | Hormonal therapy | no | yes | yes | yes |
| 8 | 47 | Black or African American | NOT Hispanic or Latino | IDC | <1% | 1% | 0% | 0% | 2+ | 1+ | Non-amplified | Non-amplified | 10 | 3 | pT1bNXMX |  | Systemic Chemotherapy, Hormonal therapy | Hormonal therapy | na | na | na | na |
| 9 | 59 | Black or African American | NOT Hispanic or Latino | IDC | <1% | 1% | <1% | 0% | 2+ | 1+ | Non-amplified | Non-amplified | 6 | 2 | pT1bN0(sn) |  | Systemic Chemotherapy; Hormone therapy | Hormonal therapy | yes | no |  | no |
| 10 | 51 | Caucasian | NOT Hispanic or Latino | IDC | 0 | 60.00% | 0 | 60.00% | 2+ | 1+ | Amplified | Non-amplified | 6 | 2 | ypT1bN0 | Systemic chemotherapy; HER2 therapy | HER2 therapy; Hormonal therapy | Hormonal therapy | yes | no |  | no |
| 11 | 68 | Black or African American | NOT Hispanic or Latino | IDC | 2% | <1% | <1% | 0% | 1+ | 0 | N/A | N/A | 7 | 2 | pT1bN0(sn) |  | None | No Hormonal therapy | no | no |  | no |
| 12 | 57 | Caucasian | NOT Hispanic or Latino | IDC | 1% | >90% | 0% | 0% | 2+ | 1+ | Non-amplified | N/A | 15 | 2 | ypT1cN0sn | Systemic Chemotherapy | Hormonal therapy | Hormonal therapy | no | no |  | no |
| 13 | 50 | Caucasian | NOT Hispanic or Latino | IDC | 5% | 0% | 0% | 0% | 3+ | 3+ | Amplified | Amplified | 15 | 3 | pT1cN0(sn) |  | Systemic chemotherapy; HER2 therapy | No hormonal therapy | yes | no |  | no |
|  |  |  |  |  |  |  |  |  |  |  |  |  |  |  |  |  |  |  |  |  |  |  |
| **HER2 Major Discrepancies** | | | | | | | | | | | | | | | | | | | | | | |
| **Case ID** | **Age** | **Race** | **Ethnicity** | **Diagnosis** | **ER- CNB** | **ER-SS** | **PR-CNB** | **PR-SS** | **HER2 IHC-CNB** | **HER2 IHC-SS** | **FISH-CNB** | **FISH-SS** | **Tumor size (mm)** | **Tumor Grade** | **Stage** | **Neoadjuvant Treatment** | **Adjuvant Treatment** | **Treatment Impact** | **Radiation** | **Recurrence** | **Metastatic disease at recurrence?** | **Death** |
| 13 |  | Black or African American | NOT Hispanic or Latino | IDC | >90% | >90% | 0% | <1% | 2+ | 3+ | Non-amplified | Amplified | 15 | 2 | pT1aN0 |  | Systemic chemotherapy; HER2 therapy; Hormone Therapy | Her2 therapy | yes | yes | no | no |
| 145 |  | Black or African American | NOT Hispanic or Latino | IDC | 0% | 0% | 0% | 0% | 2+ | 2+ | Amplified | Non-amplified | 3 | 3 | pT1aN0 |  | None | No HER2 therapy | yes | no |  | no |
| 16 |  | Black or African American | NOT Hispanic or Latino | IDC | 70% | 40% | 5% | 10% | 2+ | 3+ | Non-amplified | Amplified | 98 | 3 | pT3N1 |  | Systemic chemotherapy; HER2 therapy; Hormone Therapy | Her2 Therapy | yes | no |  | no |
| 17 |  | Caucasian | NOT Hispanic or Latino | IDC | 80% | >90% | 0% | 0% | 3+ | 2+ | Non-amplified | Non-amplified | 32 | 3 | pT2N1a |  | Systemic Chemotherapy; Hormone therapy | No HER2 therapy | no | no |  | no |
| 18 |  | Caucasian | NOT Hispanic or Latino | Mucinous | > 90% | > 90% | 10% | 1% | 1+ | 3+ | Non-amplified | Amplified | 6 | 2 | pT1bN0 |  | Systemic chemotherapy; HER2 therapy; Hormone Therapy | Her2 Therapy | yes | no |  | no |
| 19 |  | Black or African American | NOT Hispanic or Latino | IDC | 1% | 80% | 0% | <1% | 1+ | 2+ | N/A | Amplified | 16 | 3 | pT1cN1 |  | Systemic chemotherapy; HER2 therapy; Hormone Therapy | Her2 Therapy | yes | no |  | no |
| 20 |  | Caucasian | NOT Hispanic or Latino | IDC | >99% | 95% | >90% | 90% | 2+ | 3+ | Non-amplified | Amplified | 16 | 3 | pT1cN0(sn) |  | Systemic chemotherapy; HER2 therapy; Hormone Therapy | Her2 Therapy | no | no |  | no |
|  |  |  |  |  |  |  |  |  |  |  |  |  |  |  |  |  |  |  |  |  |  |  |
|  | | | | | | | | | | | | |  |  |  |  |  |  |  |  |  |  |

Abbreviations: ER: estrogen receptor; HER2: human epidermal growth factor receptor 2; IDC: invasive ductal carcinoma; ILC: invasive lobular carcinoma; N/A: not available; NA: Not available; PR: progesterone receptor
