# Supplementary material for: Concordance of breast cancer biomarker testing in core needle biopsy and surgical specimens: A single institution experience
Source: Cancer Med. 2022 Jun 22;11(24):4954–65. doi: 10.1002/cam4.4843 (PMC9761085; doi:10.1002/cam4.4843)
Supplement: Supplementary file 1 — Table S1 Table S2: Table S3: Table S4: Table S5: [file CAM4-11-4954-s001.zip › cam44843-sup-0001-TablesS1.docx]

**Supplemental Table 1:** Patient Characteristics of Cases with Major Discordance Leading to Treatment Changes

Abbreviations: ER: estrogen receptor; HER2: human epidermal growth factor receptor 2; IDC: invasive ductal carcinoma; ILC: invasive lobular carcinoma; N/A: not available; NA: Not available; PR: progesterone receptor

**Supplemental Table 2:** Treatment Changes Based on Discordance in ER, PR, HER2 IHC, and HER2 FISH in the Neoadjuvant Cohort

|  | **Major Discordance (n, %)** | **Minor Discordance (n, %)** | **P-value** |
| --- | --- | --- | --- |
| **ER (n)** | 9 | 8 | 0.08 |
| No | 4 (50.0) | 8 (100) |  |
| Yes | 4 (50.0) | 0 (0) |  |
| Missing | 1 | 0 |  |
| **PR (total n)** | 22 | 20 | >0.99 |
| No | 21 (95.5) | 18 (100) |  |
| Yes | 1 (4.6) | 0 (0) |  |
| Missing | 0 | 2 |  |
| **HER2 IHC (total n)** | 255 | 201 | NA |
| No | 25 (100.0) | 13 (100.0) |  |
| Yes | 0 (0) | 0 (0) |  |
| Missing | 0 | 0 |  |
| **HER2 FISH (total n)** | 4 | NA | NA |
| No | 0 (0) |  |  |
| Yes | 3 (100.0) |  |  |
| Missing | 1 |  |  |

Abbreviations: ER: estrogen receptor, PR: progesterone receptor, HER2: human epidermal growth factor receptor 2, IHC: immunohistochemistry, FISH: fluorescence in situ hybridization; N: number; NA: Not assessed

**Supplemental Table 3:** Recurrence, metastatic recurrence, and death in ER concordance, minor discordance, and major discordance in all patients

|  | **Concordance, N=844** | **Minor Discordance, N= 28** | **Major Discordance, N=88** | **p-value*** |
| --- | --- | --- | --- | --- |
| Recurrence, n (%) | 65 (8.1) | 7 (8.2) | 4 (15.4) | 0.38 |
| Missing | 38 | 2 | 3 |  |
| Metastatic Recurrence, n (%) | 38 (22.6) | 6 (31.6) | 3 (50.0) | 0.19 |
| Missing | 676 | 22 | 69 |  |
| Death, n (%) | 54 (6.7) | 11 (12.9) | 6 (22.2) | 0.004 |
| Missing | 37 | 1 | 3 |  |

*Fisher’s exact test

Abbreviations: ER: estrogen receptor; N: number

**Supplemental Table 4:** Recurrence, metastatic recurrence, and death in HER2 IHC concordance, minor discordance, and major discordance

|  | **Concordance, N=504** | **Minor Discordance, N= 255** | **Major Discordance, N=201** | **p-value*** |
| --- | --- | --- | --- | --- |
| Recurrence, n (%) | 43 (8.9) | 17 (6.8) | 16 (8.6) | 0.61 |
| Missing | 23 | 6 | 14 |  |
| Metastatic Recurrence, n (%) | 30 (27.3) | 7 (20.0) | 10 (20.4) | 0.53 |
| Missing | 394 | 206 | 166 |  |
| Death, n (%) | 41 (8.5) | 20 (10.6) | 10 (4.0) | 0.02 |
| Missing | 24 | 4 | 13 |  |

*Pearson’s Chi-squared test

Abbreviations: HER2: human epidermal growth factor receptor 2, IHC: immunohistochemistry; N: number

**Supplemental Table 5:** Recurrence, Metastatic Recurrence, and Death in Patients with Treatment Changes in ER and HER2 IHC Major Discordance

|  | **No Treatment Change** | **Treatment Change** | **P-value*** |
| --- | --- | --- | --- |
| **ER (n, %)** | 101 | 13 |  |
| Recurrence | 8 (8.3) | 2 (16.7) | 0.30 |
| Missing | 4 | 1 |  |
| Metastatic Recurrence | 7 (31.8) | 1 (50.0) | >0.99 |
| Missing | 79 | 11 |  |
| Death | 14 (14.3) | 2 (16.7) | 0.69 |
| Missing | 3 | 1 |  |
| **HER2 IHC (n, %)** | 450 | 6 |  |
| Recurrence | 32 (7.4) | 1 (16.7) | 0.38 |
| Missing | 20 | 0 |  |
| Metastatic Recurrence | 17 (21.0) | 0 (0) | >0.99 |
| Missing | 369 | 3 |  |
| Death | 30 (6.9) | 0 (0) | >0.99 |
| Missing | 17 | 0 |  |

* Fisher’s exact test

Abbreviations: ER: estrogen receptor, HER2: human epidermal growth factor receptor 2, IHC: immunohistochemistry
